# Efficacy and safety of levetiracetam vs. oxcarbazepine in the treatment of children with epilepsy: a systematic review and meta-analysis

**DOI:** 10.3389/fped.2024.1336744

**Published:** 2024-04-22

**Authors:** Yuanyuan Liu, Yanxu Wang, Xingzhou Li, Xiaomin Wu

**Affiliations:** ^1^ School of Public Health, Jiamusi University, Jiamusi, China; ^2^ Department of Food Hygiene Monitoring, Jiamusi City Center for Disease Control and Prevention, Jiamusi, China; ^3^ Director, Jiamusi City Center for Disease Control and Prevention, Jiamusi, China

**Keywords:** levetiracetam, oxcarbazepine, monotherapy, children with epilepsy, meta-analysis

## Abstract

**Background:**

Levetiracetam (LEV) and oxcarbazepine (OXC) are new antiseizure medications (ASMs). In recent years, OXC monotherapy is widely used in children with epilepsy; however, no consensus exists on applying LEV monotherapy among children with epilepsy.

**Objective:**

The present work focused on comparing the efficacy and safety of LEV and OXC monotherapy in treating children with epilepsy.

**Methods:**

We conducted a comprehensive search across multiple databases including PubMed, Cochrane Library, Embase, Web of Science, CNKI, Wanfang Database, VIP, and China Biology Medicine disc, covering studies from inception to August 26, 2023. We included randomized controlled trials (RCTs) and cohort studies evaluating the efficacy and safety of LEV and OXC monotherapy for treating epilepsy in children. We utilized Cochrane Risk of Bias Tool in RevMan 5.3 software for assessing included RCTs quality. In addition, included cohort studies quality was determined using Newcastle-Ottawa Scale (NOS). A random-effects model was utilized to summarize the results.

**Results:**

This meta-analysis included altogether 14 studies, including 893 children with epilepsy. LEV and OXC monotherapy was not statistical different among children with epilepsy in seizure-free rate (relative risk [RR] = 1.010, 95% confidence interval [CI] [0.822, 1.242], *P *> 0.05) and seizure frequency decrease of ≥50% compared with baseline [RR = 0.938, 95% CI (0.676, 1.301), *P *> 0.05]. Differences in total adverse reaction rate [RR = 1.113, 95% CI (0.710, 1.744), *P *> 0.05] and failure rate because of serious adverse reaction [RR = 1.001, 95% CI (0.349, 2.871), *P *> 0.05] were not statistical different between LEV and OXC treatments among children with epilepsy. However, the effects of OXC monotherapy on thyroid among children with epilepsy was statistically correlated than that of LEV (thyroid stimulating hormone: standardized mean difference [SMD] = −0.144, 95% CI [−0.613, 0.325], *P *> 0.05; free thyroxine: SMD = 1.663, 95% CI [0.179, 3.147], *P *< 0.05).

**Conclusion:**

The efficacy of LEV and OXC monotherapy in treating children with epilepsy is similar. However, OXC having a more significant effect on the thyroid than that of LEV. Therefore, LEV may be safer for children with epilepsy who are predisposed to thyroid disease than OXC.

**Systematic Review Registration:**

https://www.crd.york.ac.uk/, PROSPERO (CRD42024514016)

## 1 Introduction

Epilepsy represents the commonly occurring chronic brain disease causing mortality in approximately 125,000 individuals annually (1). It is characterized by recurrent seizures, and children's brains are not completely developed; repeated seizures often cause critical neurological damage, and can lead to intellectual disability in severe cases (2). Based on the statistics, the global incidence of childhood epilepsy is 41–187/100,000; and its global prevalence is higher than its incidence. To be specific, the prevalence of childhood epilepsy in developed and less developed countries is 3.2–5.5/1,000 and 3.6–44/1,000, respectively (3). Epilepsy in children is primarily treated by antiseizure medications (ASMs). ASMs have exhibited good efficacy in approximately 70% of pediatric patients (4).

Since 1990, more than 20 novel ASMs have been introduced, with similar efficacy but better safety compared to traditional ASMs (5). A novel ASM, oxcarbazepine (OXC), is a derivative that improves the safety and pharmacokinetic characteristics of carbamazepine (CBZ) and reduces the interaction between CBZ and other ASMs (6). OXC's mechanism of action blocks sodium channels and controls the abnormal firing of neurons (7). Another novel ASM, levetiracetam (LEV), demonstrates the antiepileptic mechanism of action through combination with synaptic vesicle protein SV2A, interference with neurotransmitter release in vesicles, control of rapid firing of neurons, inhibition of Ca^2+^ release and blockage of Ca^2+^ channels, and control of the excessive synchronization between neurons (8). It is advantageous owing to its high bioavailability, linear pharmacokinetics, low plasma protein binding, no liver metabolism, renal excretion, and good tolerance (9).

In recent times, monotherapy with OXC has gained widespread use in treating childhood epilepsy. Nevertheless, there is no consensus regarding the utilization of LEV as a monotherapy in this same patient population. OXC has received global registration in over 50 countries. OXC monotherapy is extensively employed in numerous nations, such as the United States, China, and Europe, to manage epilepsy in children (10–12). While LEV has been granted approval for monotherapy use in children with epilepsy in China, it has only been sanctioned for monotherapy for this purpose in European children aged ≥16 years. Notably, it has not received approval for treating children with epilepsy as a monotherapy in the USA (13). The 2018 American Academy of Neurology Guidelines and recommendations from Belgian epilepsy experts in 2020 advise OXC being used alone or as adjuvant treatment for childhood epilepsy, whereas LEV is suggested for adjunctive treatment (14, 15).

Systematic reviews and meta-analyses of mixed ASMs types, which encompass not only LEV and OXC but also various other ASMs, has revealed that LEV and OXC are comparable in terms of the efficacy and safety on epilepsy in pediatric patients (16, 17). Nevertheless, recent studies published within the last five years have produced varying results concerning both the efficacy and safety of these medications. Zhao et al. (18) indicated that OXC monotherapy was more high than LEV on treating focal epileptic infants in seizure freedom and the 12-month retention rate. Shi et al. (19) demonstrated that, in the context of childhood epilepsy, OXC monotherapy was more associated with adverse effects on thyroid function and bone metabolic disturbances when compared to LEV monotherapy.

Additionally, when it comes to systematic reviews and meta-analyses of mixed ASMs types, there is a scarcity of literature comparing the efficacy and safety of LEV and OXC treatments alone in treating epilepsy in pediatric patients. Only a maximum of six articles available, and have not provided comparison between LEV and OXC treatments alone on managing childhood epilepsy, in terms of failure rate because of serious adverse reaction, and effects on the thyroid gland. Therefore, this study has encompassed data from 14 different studies involving 893 participants to provide a comprehensively compare the efficacy and safety of LEV and OXC monotherapy in the treatment of childhood epilepsy. This endeavor seeks to offer updated and more dependable evidence to inform the clinical application of LEV treatment alone in treating childhood epilepsy.

## 2 Methods

### 2.1 Study screening procedure

Our systematic review and meta-analysis followed the Preferred Reporting Items for Systematic Reviews and Meta-Analyses (PRISMA) guidelines (20). Our research protocol has been registered on the International Prospective Register of Systematic Reviews (PROSPERO) (CRD42024514016). Four English databases (PubMed, Cochrane Library, Embase, Web of Science) together with four Chinese databases (China National Knowledge Infrastructure [CNKI], Wanfang Database, China Science and Technology Journal Database [VIP], China Biology Medicine disc), were comprehensively searched to identify published studies examining the efficacy and safety of LEV and OXC treatments alone in treating pediatric epilepsy from their inception to August 26, 2023. Search terms included “Levetiracetam”, “Keppra”, “Oxcarbazepine”, “Trileptal”, “Child”, “Children”, “Infant”, “Adolescent”, “Pediatrics”, “Epilepsy”, and “Seizure Disorder.” Supplementary Appendix S1 displays detailed results regarding PubMed search strategy. Additionally, relevant reviews, systematic reviews, meta-analyses from last three years, as well as references from the included studies, were manually scrutinized to avoid overlooking qualified studies.

### 2.2 Inclusion and exclusion criteria

Studies below were included: (1) Participants: Children with epilepsy under 18 years, irrespective of epilepsy type, gender, ethnicity, or geographic location. (2) Interventions: The LEV and OXC groups received LEV and OXC monotherapies, respectively, without restrictions on dosage form, dosage, route of administration, frequency, or treatment duration. (3) Outcomes: seizure-free rate (children with epilepsy treated with LEV and OXC monotherapy had no more seizures or breakthrough seizures only with missed doses of medication), seizure frequency decrease of ≥50% compared with baseline (seizure frequency decrease of ≥50% compared with baseline in children with epilepsy treated with LEV and OXC monotherapy), total adverse reaction rate (the total number of adverse effects observed in children with epilepsy treated with LEV and OXC monotherapy), failure rate because of serious adverse reaction (defined as adverse effects leading to the addition of a second ASM, change to another ASM, or discontinuation of ASM treatment), and effects on the thyroid (thyroid stimulating hormone[TSH] and free thyroxine [fT4] levels before and after LEV and OXC monotherapy in children with epilepsy). One or more primary and secondary outcome indicators must be reported. (4) Study Types: Randomized controlled trials (RCTs) and cohort studies conducted in both Chinese and English languages.

Studies below were excluded: (1) Duplicates. (2) Reviews, systematic reviews, meta-analyses, guidelines, commentaries, conference abstracts, case reports, letters, and animal studies. (3) Unavailability of full-text, unpublished literatures. (4) Incorrect or incomplete data, or inability to provide data that can be transformed into aggregated data. (5) Chinese literatures not published in Peking University's “A Guide to the Core Journal of China.”

### 2.3 Study selection and data collection

Those searched articles were imported in EndNote 20 software. Two researchers were responsible for study selection and data collection according to inclusion and exclusion criteria. A third researcher assisted in making the final decision for any disputed sections. Using Excel 2019, data below were collected, first author, publication year, country, study type, sample size per group, sex, age, therapeutic time, and outcome.

### 2.4 Study quality assessment

We utilized Cochrane Risk of Bias Tool (21) in RevMan 5.3 software for assessing included RCTs quality. In addition, included cohort studies quality was determined using Newcastle-Ottawa Scale (NOS) (22). The quality assessment process was performed independently by two researchers. A third researcher assisted in resolving any disputes. Seven items were included in Cochrane Risk of Bias Assessment Tool: random sequence generation, allocation concealment, blinding of participants and personnel, blinding of outcome assessment, incomplete outcome data, selective reporting, together with other bias. Besides, every item was classified into unclear, low, or high bias risk. NOS consisted of three sections: selection, comparability, and outcome, its total score was 9 and ≥6 scores indicated high-quality studies.

### 2.5 Statistical analysis

Stata 15.1 software was employed for statistical analysis. Dichotomous variables were analyzed by relative risk (RR) together with 95% confidence intervals (95% CIs) for effect sizes, and *P* < 0.05 stood for statistical significance. Continuous variables were analyzed by standardized mean difference (SMD) and 95% CIs for effect sizes, and *P* < 0.05 stood for statistical significance. At least three original studies for each outcome indicator were combined. Continuous variables were combined directly using post-treatment in the absence of significant difference before treatment; otherwise, changes before and after treatment were combined. Effect sizes were combined with random-effects models.

## 3 Results

### 3.1 Study selection results and basic study characteristics

**Figure 1:**
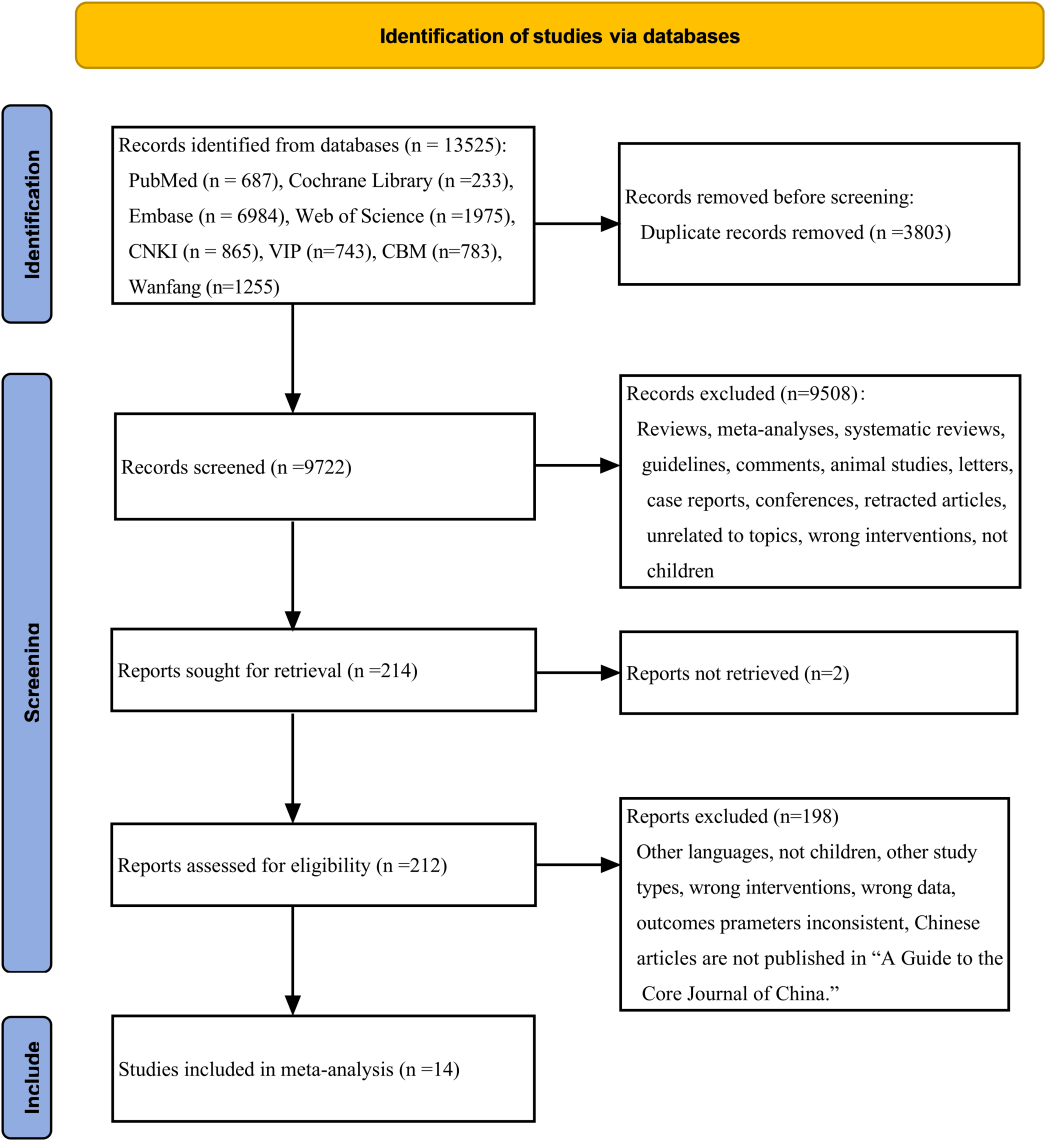
Flow diagram of study selection process.

13,525 studies were initially identified. After duplicate removal and thorough title-, abstract- and full-text-reading, we ultimately chose 14 articles for this meta-analysis. This specific process is outlined in Figure 1. This comprised 893 children with epilepsy, with 465 of them undergoing LEV treatment and 428 receiving OXC. The selected literature ranged from 2007 to 2023 and encompassed six RCTs (19, 23–27) and eight cohort studies (18, 28–34). Among these, eight studies reported on the efficacy (18, 23, 25–27, 29, 31, 34), while nine studies presented data on safety (19, 23, 24, 28, 30–34). The required details about those enrolled articles are displayed in Table 1.

**Table 1:** Basic characteristics of enrolled studies.

Study (Author, year)	Country	Study type	Sample size (*n*)		Sex (M/F, *n*)		Age(mean ± SD or range)		Dosage		Treatment time (months)	Outcomes
			LEV	OXC	LEV	OXC	LEV	OXC	LEV	OXC		
Giangennaro Coppola 2007 (23)	Italy	RCT	21	18	11/10	10/8	5–13 years	3.3–14 years	Initial dose: 5 mg/kg/d, maximum dose: 20–30 mg/kg/d	Initial dose: 5 mg/kg/d, maximum dose: 20–35 mg/kg/d	12–24	①③④
Guihai Suo 2021 (24)	China	RCT	32	32	21/11	19/13	8.47 ± 2.13 years	8.62 ± 2.21 years	Initial dose: 10 mg/kg/d, maintenance dose: 20–60 mg/kg/d	Initial dose: 8–10 mg/kg/d, maintenance dose: 20–46 mg/kg/d	6	③
Kaili Shi 2020 (19)	China	RCT	23	20	14/9	12/8	7.69 ± 2.68 years	7.70 ± 2.95 years	Initial dose: 10 mg/kg/d, maintenance dose: 30–40 mg/kg/d	Initial dose: 5–10 mg/kg/d, maintenance dose: 20–40 mg/kg/d	12	⑤⑥
Yanmin Bai 2023 (25)	China	RCT	28	28	11/17	15/13	42.6 ± 9.8 months	43.2 ± 11.3 months	Initial dose: 10–20 mg/kg/d, if not improved, can be increased after 1 week, each increase of 10–20 mg/kg/d	Initial dose: 8–10 mg/kg/d, if not improved, can be increased after 12 week, each increase of 10 mg/kg/d, maximum dose: ≤45 mg/kg/d	6	①②
Bo Jin 2013 (26)	China	RCT	20	17	NR	NR	53–152 months	53–152 months	Initial dose: 20 mg/kg/d, maximum dose: 60 mg/kg/d	Initial dose: 8 mg/kg/d, maximum dose: 42 mg/kg/d	3–38	①②
Yakun Du 2019 (27)	China	RCT	50	50	32/18	28/22	6.2 ± 3.7 years	6.8 ± 4.0 years	Initial dose: 10 mg/kg/d, maintenance dose: 20–40 mg/kg/d	Initial dose: 5–10 mg/kg/d, maintenance dose: 20–30 mg/kg/d	3–6	①②
Ünsal Yılmaz 2014 (28)	Turkey	Cohort study	11	14	4/7	8/6	1–18 years	1–18 years	NR	NR	12	⑤⑥
Binyang Zhao 2022 (18)	China	Cohort study	78	83	30/48	38/45	2–24 months	2–24 months	Initial dose: 10 mg/kg/d, maintenance maximal dose: 40 mg/kg/d	Initial dose: 10 mg/kg/d, maintenance maximal dose: 40 mg/kg/d	12	①
Francesca Felicia Operto 2020 (29)	Italy	Cohort study	46	20	NR	NR	11 ± 2.6 years	10 ± 2.4 years	1,033 ± 205 mg/d	1,050 ± 392 mg/d	9	①②
Reem A. Abdel Aziz 2018 (30)	Egypt	Cohort study	20	20	11/9	13/7	4.7 ± 2.2 years	5.6 ± 2.5 years	21.4 ± 3 (16–30)	12 ± 3.4 (10–20)	6	⑤⑥
Ünsal Yılmaz 2014 (31)	Turkey	Cohort study	34	38	13/21	21/17	1–18 years	1–18 years	NR	NR	12	①③④
Fatih Aygün 2012 (32)	Turkey	Cohort study	5	6	NR	NR	3–168 months	3–168 months	Mean daily dosage: 50 mg/kg/d	Mean daily dosage: 30 mg/kg/d	9	⑤⑥
Astrid Bertsche 2014 (33)	Germany	Cohort study	42	34	NR	NR	0.5–16.7 years	1.9–16.9 years	minimum dose: 27.1 mg/kg/d, maximum dose: 108.0 mg/kg/d	minimum dose: 10.7 mg/kg/d, maximum dose: 71.0 mg/kg/d	12	④
Tao Chen 2013 (34)	China	Cohort study	55	48	NR	NR	9.8 ± 3.5 years	9.8 ± 3.5 years	Initial dose: 20 mg/kg/d, maintenance dose: 30–40 mg/kg/d	Initial dose: 5–10 mg/kg/d, maintenance dose: 20–40 mg/kg/d	6	①②③

LEV, levetiracetam; OXC, oxcarbazepine; RCT, randomized controlled trial; NR, not reported; ① Seizure-free rate; ② Seizure frequency decrease of ≥50% compared with baseline; ③ Total adverse reaction rate; ④ Failure rate because of serious adverse reaction; ⑤ Thyroid stimulating hormone (TSH); ⑥ free thyroxine (fT4).

### 3.2 Quality evaluation of the literature

**Figure 2:**
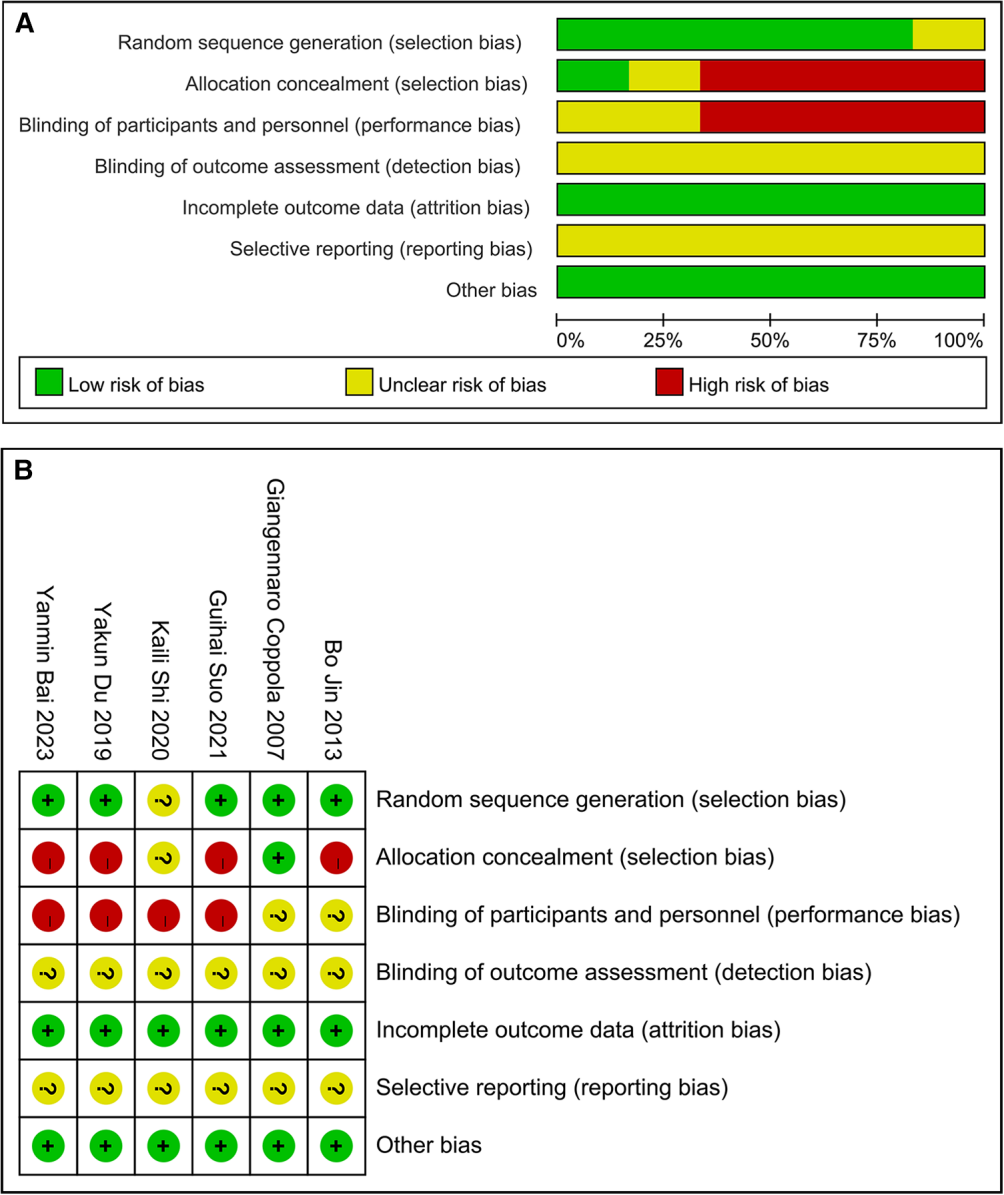
Quality assessment of RCTs. (**A**) A graph showing bias risk. (**B**) Summary of bias risk.

The assessment of the quality of six RCTs revealed that in five of them, generation of random sequence and in one, concealment of allocation showed a low bias risk. In four RCTs, a high bias risk was detected in blinding participants and personnel, while in two, there remained an unclear bias risk. All the articles had an unclear bias risk regarding blinding of outcome assessment. All the articles had a low bias risk regarding incomplete outcome data. All the articles had an unclear bias risk regarding selective reporting. Furthermore, in these articles, other bias risk showed a low bias risk. These findings can be observed from Figure 2.

Quality evaluation for the eight cohort studies demonstrated one, three, and four studies with a total score of eight, seven, and six (Table 2).

**Table 2:** Quality evaluation for the cohort studies [Newcastle Ottawa scale (NOS)].

Study	Year	Selection	Comparability	Outcome	Total
Ünsal Yılmaz	2014	3	1	3	7
Binyang Zhao	2022	4	2	2	8
Francesca Felicia Operto	2020	3	2	2	7
Reem A. Abdel Aziz	2018	3	1	2	6
Ünsal Yılmaz	2014	4	1	2	7
Fatih Aygün	2012	3	1	2	6
Astrid Bertsche	2014	3	1	2	6
Tao Chen	2013	3	1	2	6

### 3.3 Meta-analysis results

#### 3.3.1 Efficacy outcomes

##### 3.3.1.1 Seizure-free rate

**Figure 3:**
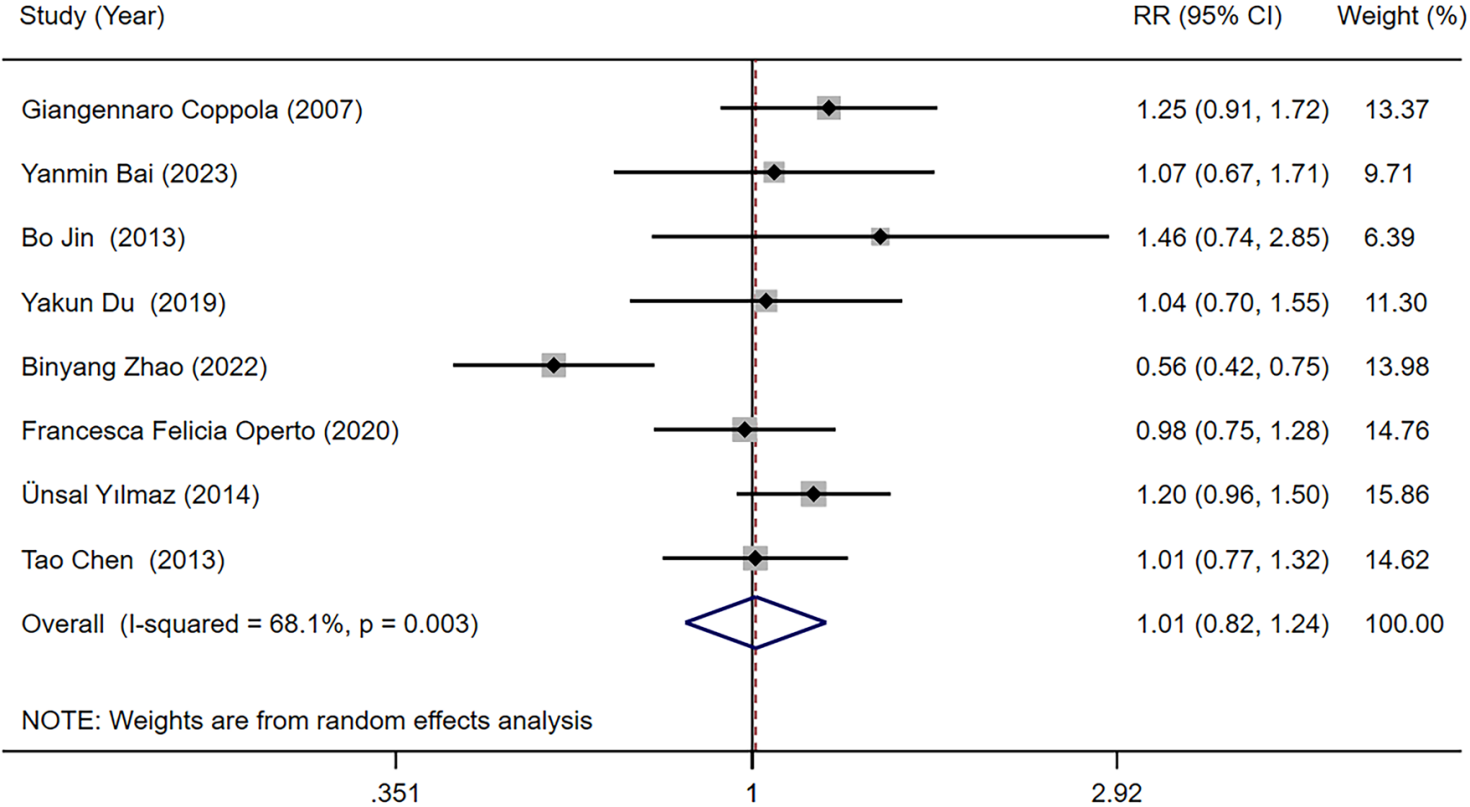
A forest plot of the seizure-free rate of levetiracetam (LEV) vs. oxcarbazepine (OXC).

**Figure 4:**
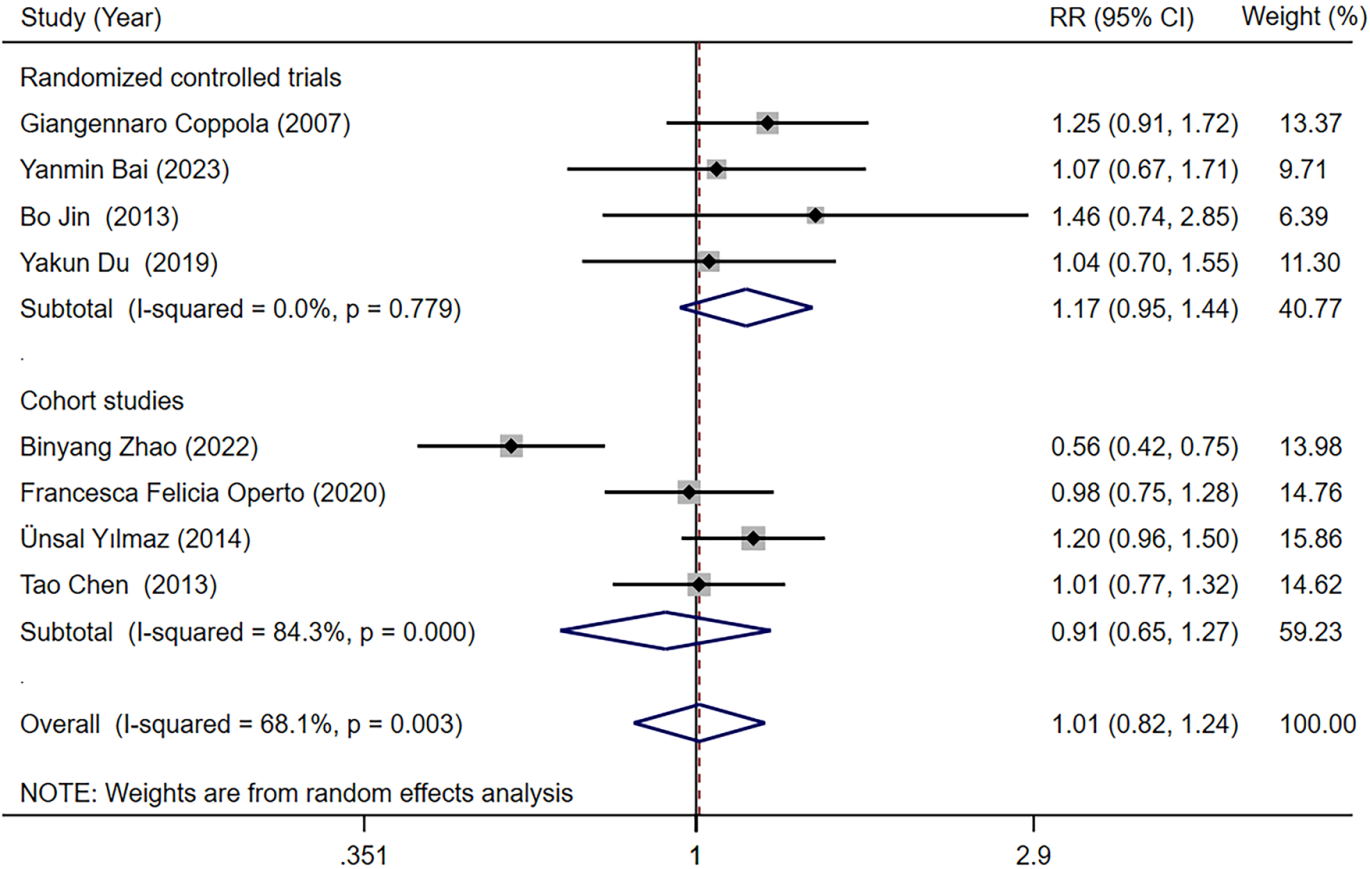
A forest plot of subgroup analysis for the seizure-free rate of levetiracetam (LEV) vs. oxcarbazepine (OXC).

Eight studies (18, 23, 25–27, 29, 31, 34) reported the seizure-free rate in LEV and OXC treatments alone for children with epilepsy. Our meta-analysis results demonstrated that seizure-free rate [RR = 1.010, 95% CI (0.822, 1.242), *P* = 0.923 > 0.05] was not significantly different between two treatments (Figure 3). We conducted subgroup analysis based on study type, which indicated no statistically difference in seizure-free rate between LEV and OXC in the treatment of childhood epilepsy, either in RCTs or cohort studies (RCTs, RR = 1.171, 95% CI [0.950, 1.443], *P* = 0.139 > 0.05; cohort studies, RR = 0.908, 95% CI [0.651, 1.266], *P* = 0.569 > 0.05) (Figure 4).

##### 3.3.1.2 Seizure frequency decrease ≥50% compared with baseline

**Figure 5:**
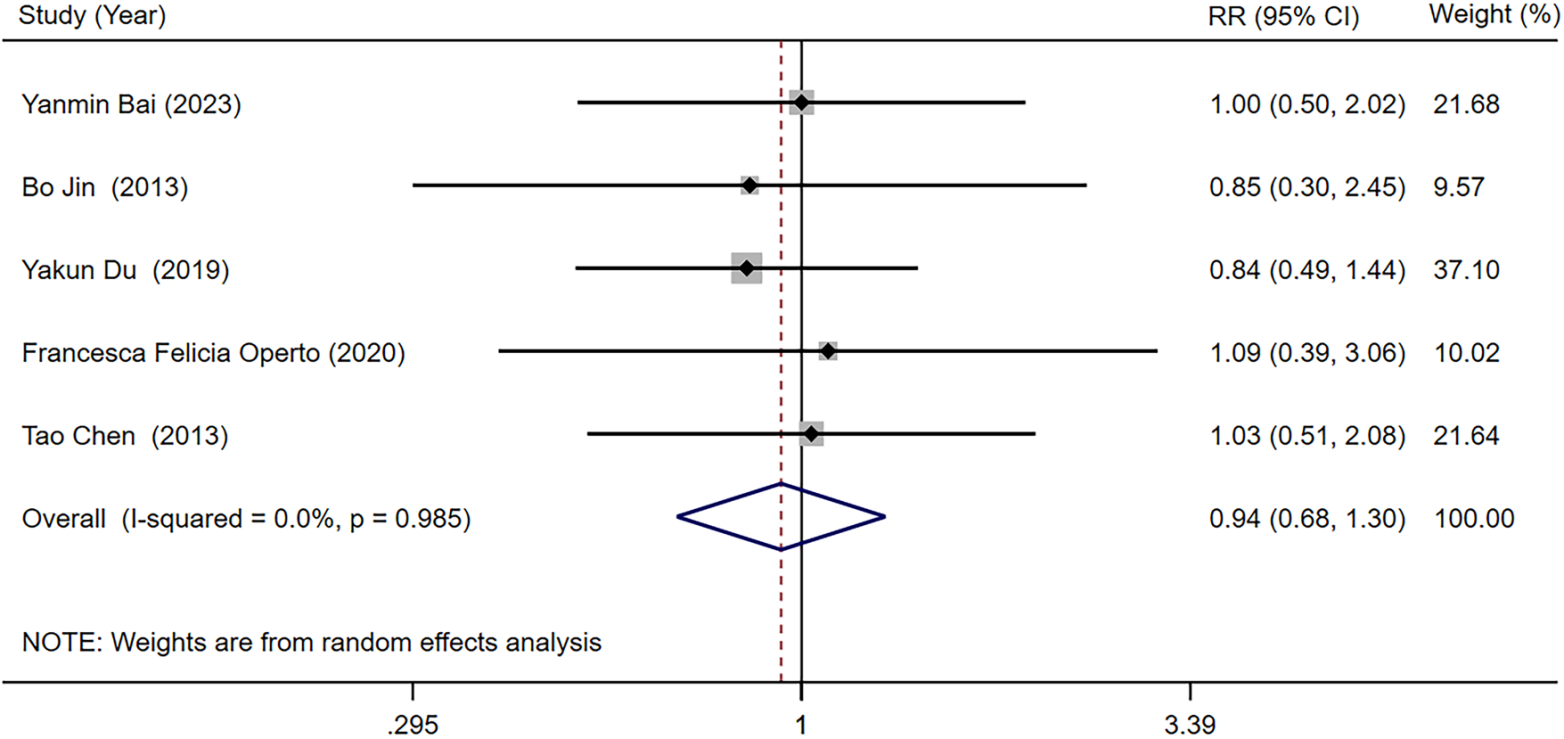
A forest plot of the seizure frequency decrease ≥50% compared with baseline of levetiracetam (LEV) vs. oxcarbazepine (OXC).

Five studies (25–27, 29, 34) reported the seizure frequency decrease ≥50% compared with baseline in LEV and OXC treatments alone for children with epilepsy. Our meta-analysis results suggested that seizure frequency decrease ≥50% compared with baseline was not significantly different between two treatments [RR = 0.938, 95% CI (0.676, 1.301), *P* = 0.700 > 0.05] (Figure 5).

#### 3.3.2 Safety outcomes

##### 3.3.2.1 Total adverse reaction rate

**Figure 6:**
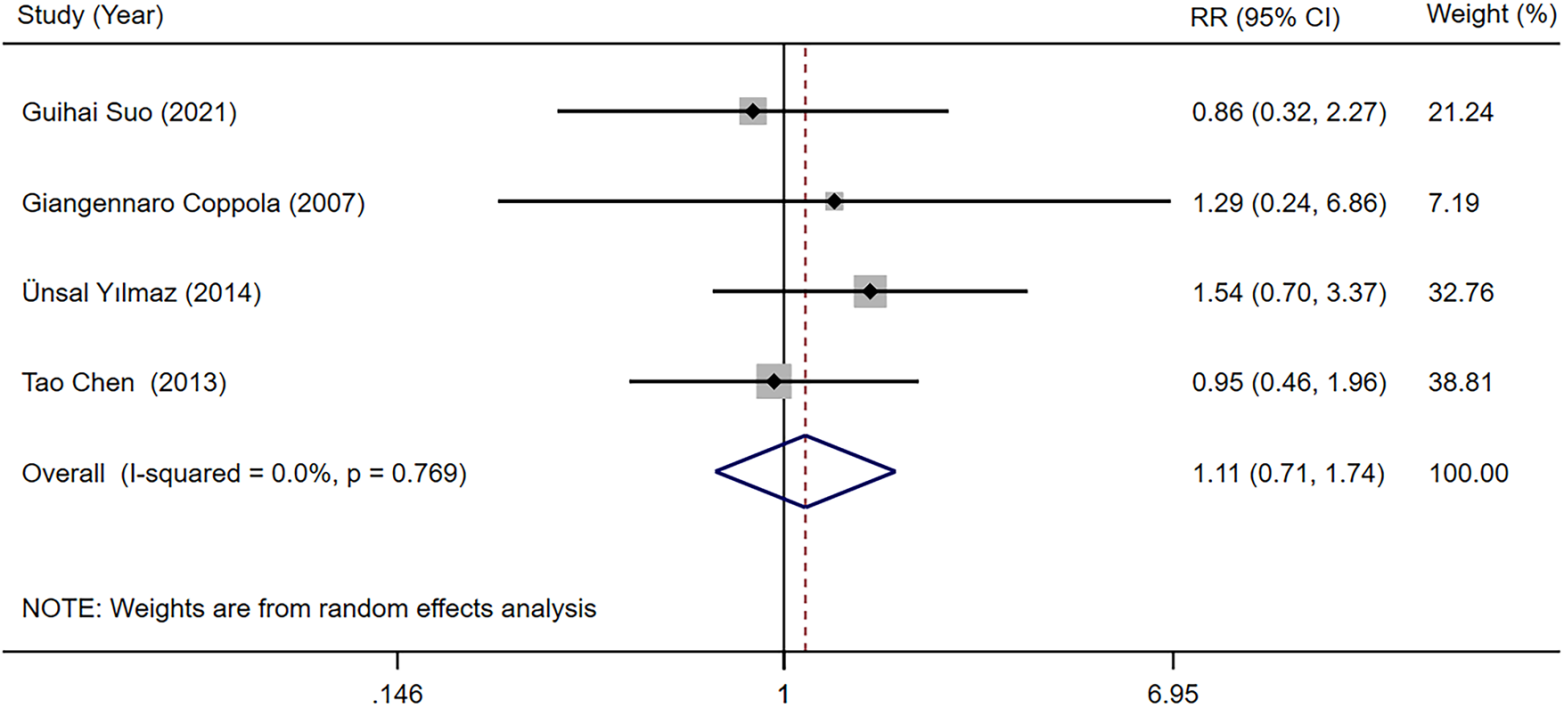
A forest plot of the total adverse reaction rate of levetiracetam (LEV) vs. oxcarbazepine (OXC).

Four studies (23, 24, 31, 34) provided information regarding total adverse reaction rate in children with epilepsy receiving LEV and OXC monotherapy. Our meta-analysis outcomes suggested that total adverse reaction rate was not significantly different between two treatments [RR = 1.113, 95% CI (0.710, 1.744), *P* = 0.640 > 0.05] (Figure 6).

##### 3.3.2.2 Failure rate because of serious adverse reaction

**Figure 7:**
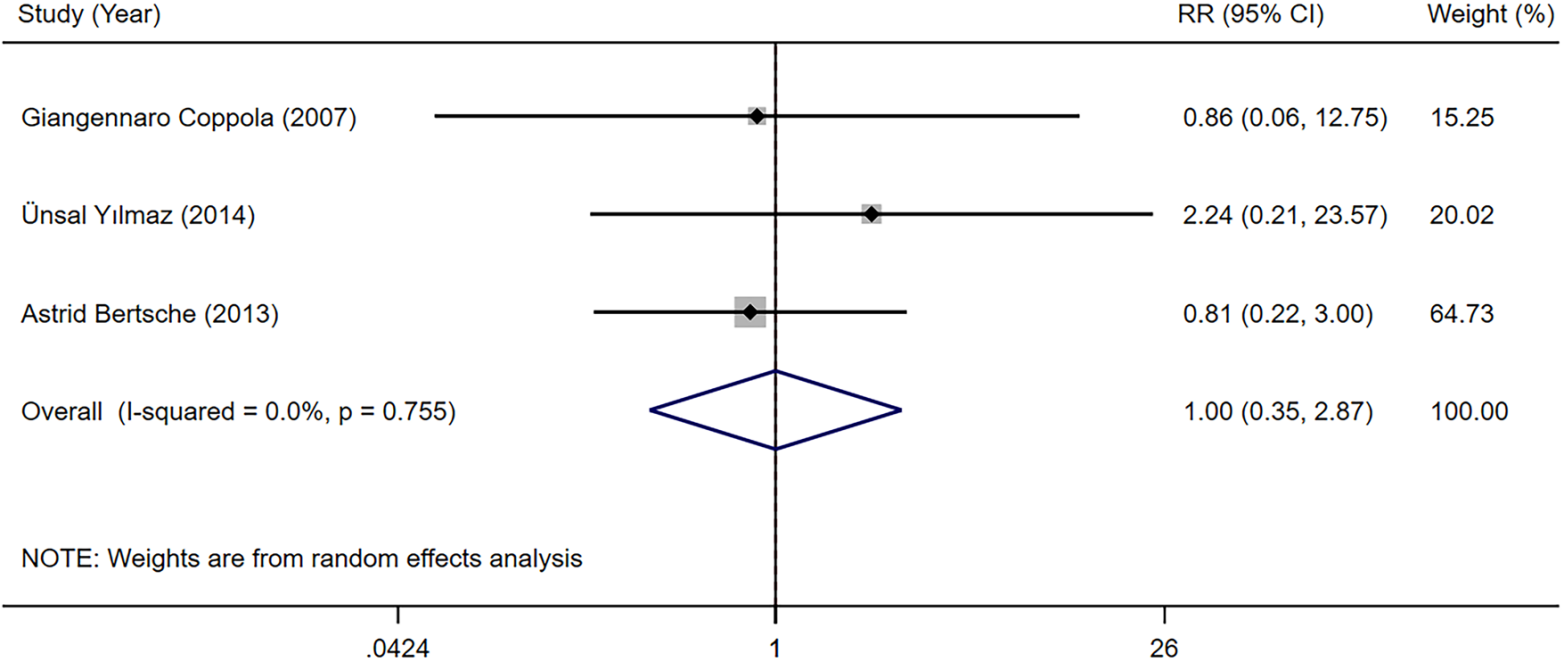
A forest plot of the failure rate because of serious adverse reaction of levetiracetam (LEV) vs. oxcarbazepine (OXC).

Three studies (23, 31, 33) provided data on the failure rate because of serious adverse reaction among children with epilepsy undergoing LEV and OXC monotherapy. Our meta-analysis results indicated that the failure rate because of serious adverse reaction was not significantly different between two groups [RR = 1.001, 95% CI (0.349, 2.871), *P* = 0.999 > 0.05] (Figure 7).

##### 3.3.2.3 Effects on the thyroid gland

**Figure 8:**
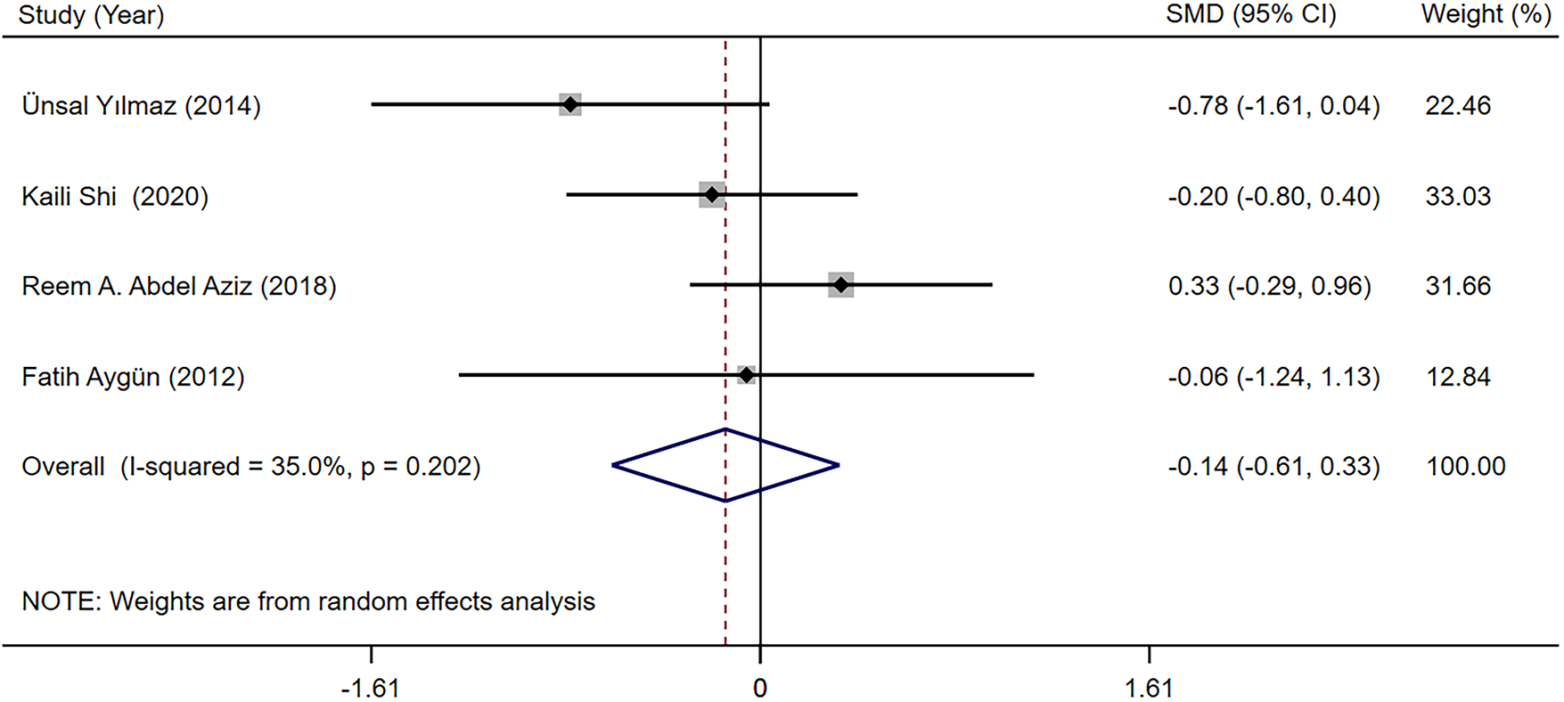
A forest plot of the effect on thyroid stimulating hormone (TSH) levels of levetiracetam (LEV) vs. oxcarbazepine (OXC).

**Figure 9:**
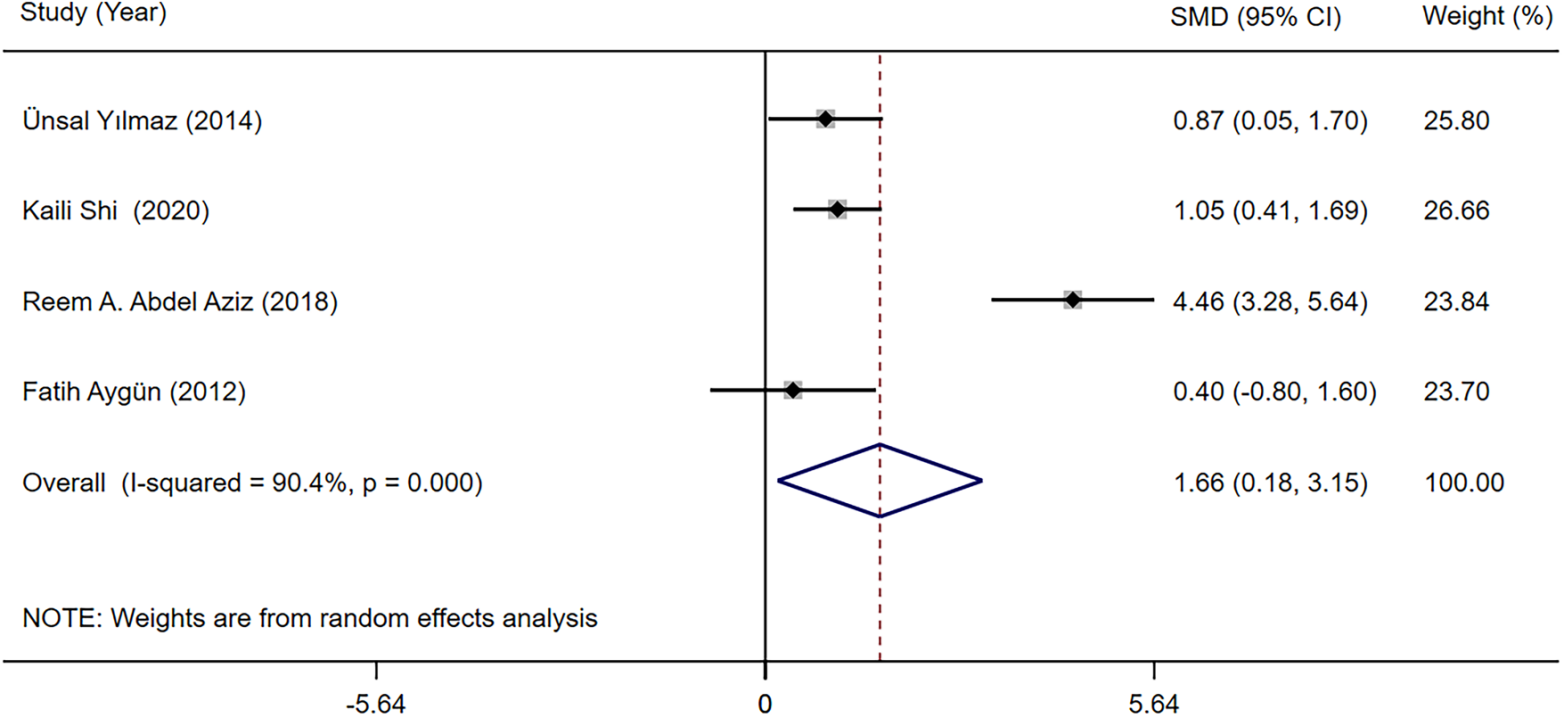
A forest plot of the effect on free thyroxine (fT4) levels of levetiracetam (LEV) vs. oxcarbazepine (OXC).

The TSH and fT4 levels in children with epilepsy before and after LEV and OXC monotherapy were reported in four studies (19, 28, 30, 32). According to our meta-analysis finding, TSH and fT4 levels were not significantly different between LEV and OXC before treatment (Supplementary Appendix S2, S3). Therefore, the levels of TSH and fT4 after treatment were directly combined. According to our meta-analysis finding, the effects of LEV and OXC on TSH was not significantly different [SMD = −0.144, 95% CI (−0.613, 0.325), *P* = 0.548 > 0.05] (Figure 8). However, OXC-reduced fT4 levels were statistically correlated than that of LEV [SMD = 1.663, 95% CI (0.179, 3.147), *P* = 0.028 < 0.05] (Figure 9).

### 3.4 Sensitivity analysis and publication bias

**Figure 10:**
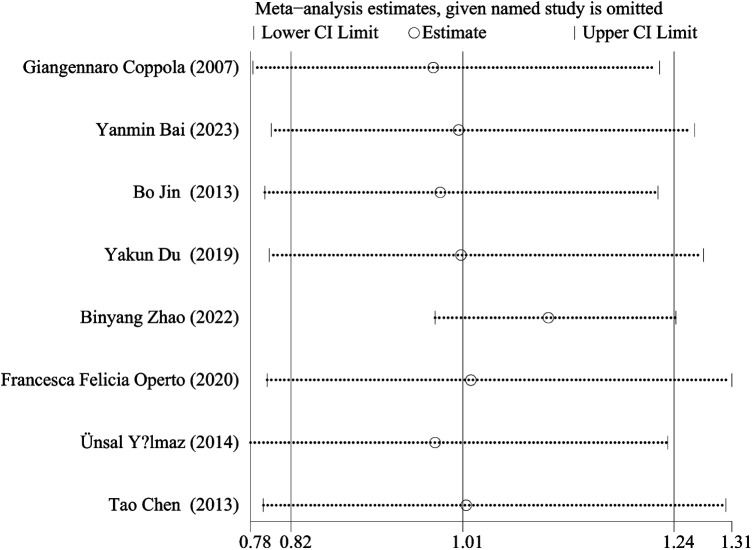
Sensitivity analysis.

We performed a sensitivity analysis by comparing the results of the meta-analysis after the exclusion of each study with the results of the meta-analysis before the exclusion. The results showed that there was no statistical difference in seizure free rate between LEV and OXC monotherapy in children with epilepsy remained stable and reliable (Figure 10).

A minimum of 10 studies should be included to use the funnel plot asymmetry test, based on Cochrane Handbook for Systematic Reviews of Interventions (35), and if too few studies are included, the efficacy of the test will be too low, failing to truly differentiate between symmetry or not. Therefore, we did not assess publication bias in the present work.

## 4 Discussion

Our systematic review and meta-analysis yielded the following insights: (1) Seizure-free rate and seizure frequency decrease ≥50% compared with baseline were not significantly different in children with epilepsy when comparing LEV and OXC monotherapy. (2) Total adverse reaction rate, failure rate because of severe adverse reaction, between LEV and OXC treatments alone were not significantly different. However, the effects of OXC on the thyroid was greater than that of LEV.

LEV and OXC are considered as new ASMs. LEV is primarily utilized to be adjuvant treatment in children with epilepsy, whereas OXC is applied as monotherapy or adjuvant treatment in pediatric epilepsy. Numerous studies have indicated that OXC, when employed as a monotherapy or adjunctive treatment for childhood epilepsy, can yield favorable outcomes (36–38). We compared the efficacy of LEV and OXC monotherapy in children with epilepsy, and our results indicated that there were no statistical differences in seizure-free rate and seizure frequency decrease of ≥50% compared with baseline. Some prior studies have reported results consistent with our findings. Geng et al. (16) discovered that seizure-free rates and seizure frequency decrease of more than 75%, 50%–75%, or less than 50% compared with baseline were not significantly different between OXC and LEV monotherapies among children with epilepsy. Similarly, Zhang et al. (17) suggested that seizure-free rate and seizure frequency decrease ≥50% compared with baseline were not significantly different when comparing LEV and OXC monotherapy among children with epilepsy. Coppola et al. (23) also observed that seizure-free rate was not significantly different in LEV vs. OXC treatment alone for children with benign epilepsy with centrotemporal spikes.

ASMs-related adverse reactions notably affect patient life quality and treatment adherence among individuals with epilepsy, potentially leading to discontinuation of therapy (39). In this study, the incidence of total adverse reaction for LEV and OXC was 22.53% and 20.59%, respectively. Both LEV and OXC displayed a favorable safety profile, with the majority of adverse effects being mild. Nonetheless, some serious adverse reactions, including headache, behavioral and emotional disturbances, diplopia, and rashes, resulted in treatment failure. Our findings revealed no statistical difference in total adverse reaction rate, and failure rate because of serious adverse reaction, in LEV vs. OXC when treating children with epilepsy. These results align with some previous studies. For instance, Suo et al. (24) demonstrated that LEV and OXC monotherapies were not significantly different with regard to total adverse reaction rate, among children with benign epilepsy with centrotemporal spikes. Bertsche et al. (33) found that the treatment failure rate because of adverse drug reaction was not significantly different between LEV vs. OXC monotherapy in children with focal epilepsy. Therefore, it is essential for children with epilepsy receiving LEV or OXC for initiating treatment with a low dose, progressively increase the dosage, monitor blood drug concentrations as needed, and maintain close follow-up to promptly detect and address adverse effects as they arise.

Thyroid hormones have a crucial effect on central nervous system development, normal physiological functions of the brain, and repair mechanisms (40). Even minor alterations in thyroid hormones, including subclinical hypothyroidism, can hinder growth and development in children (41). ASMs can influence thyroid hormone biosynthesis, production, transportation, metabolism, and excretion, causing varying degrees of harm to in thyroid-hormone homeostasis (42). Thyroid irregularities have been reported in one-third of epilepsy patients taking ASMs (43). Our research found no statistical notable difference in TSH levels between LEV and OXC monotherapies among children with epilepsy. However, OXC is linked to more reduction in fT4 levels compared to LEV. Some prior studies have reported findings in line with our study. For instance, Yılmaz et al. (28) revealed that serum fT4 levels did not significantly change after 12 months of LEV monotherapy among children with epilepsy, and the incidence of subclinical hypothyroidism was 0%. In contrast, the level of fT4 decreased after 12 months of OXC monotherapy, with an incidence rate of 21.4% for subclinical hypothyroidism. Aziz et al. (30) suggested that TSH and fT4 levels were not significantly changed after LEV treatment for over 6 months in children with epilepsy. They noted that TSH level were not significantly changed when treating childhood epilepsy with OXC for over 6 months. However, fT4 levels decreased. Therefore, it is advisable to closely monitor thyroid function in children with epilepsy who are administered OXC. For children with epilepsy prone to thyroid issues, OXC treatment should be approached with caution.

This systematic review and meta-analysis have some limitations: (1) We included 14 studies, but some had relatively small sample sizes, potentially affecting result accuracy. (2) The limited number of included studies makes it challenging to compare the efficacy and safety of LEV and OXC monotherapies in children across different age groups. (3) There was heterogeneity due to differences in epilepsy diagnostic criteria, epilepsy type, dosage, and treatment duration, which may weaken the strength of the evidence. (4) The lack of uniform criteria and quantitative evaluation for adverse reactions of LEV and OXC, coupled with the limited number of studies included, pose challenges in gathering comprehensive data on adverse reactions of LEV and OXC monotherapy in the treatment of children with epilepsy. Hence, additional large-sample, high-quality RCTs should be conducted for confirming efficacy and safety of LEV compared to OXC in treating children with epilepsy.

## 5 Conclusion

To sum up, the present systematic review and meta-analysis indicate that LEV and OXC monotherapies achieve comparable efficacy in treating childhood epilepsy. However, the effects of OXC on the thyroid was greater than that of LEV. As a result, LEV may be a preferable choice for children with epilepsy who have a predisposition to thyroid issues.

## Data Availability

The original contributions presented in the study are included in the article/**Supplementary Material**, further inquiries can be directed to the corresponding authors.
