# Supplementary material for: Efficacy and safety of levetiracetam vs. oxcarbazepine in the treatment of children with epilepsy: a systematic review and meta-analysis
Source: Front Pediatr. 2024 Apr 22;12:1336744. doi: 10.3389/fped.2024.1336744 (PMC11072191; doi:10.3389/fped.2024.1336744)
Supplement: Supplementary file 2 [file Datasheet2.pdf]

Supplementary Material

Efficacy and safety of levetiracetam vs. oxcarbazepine in the treatment of children with epilepsy: a systematic review and meta-analysis

Yuanyuan Liu, Yanxu Wang, Xingzhou Li*†, Xiaomin Wu*†

*** Correspondence:** Xingzhou Li: lxzhwhd0454@126.com Xiaomin Wu: wxm5805@126.com

† These authors have contributed equally to this work.

1 Supplementary Appendix S1

Supplementary Appendix S1. Detailed results regarding PubMed search strategy.

2 Supplementary Appendix S2, S3

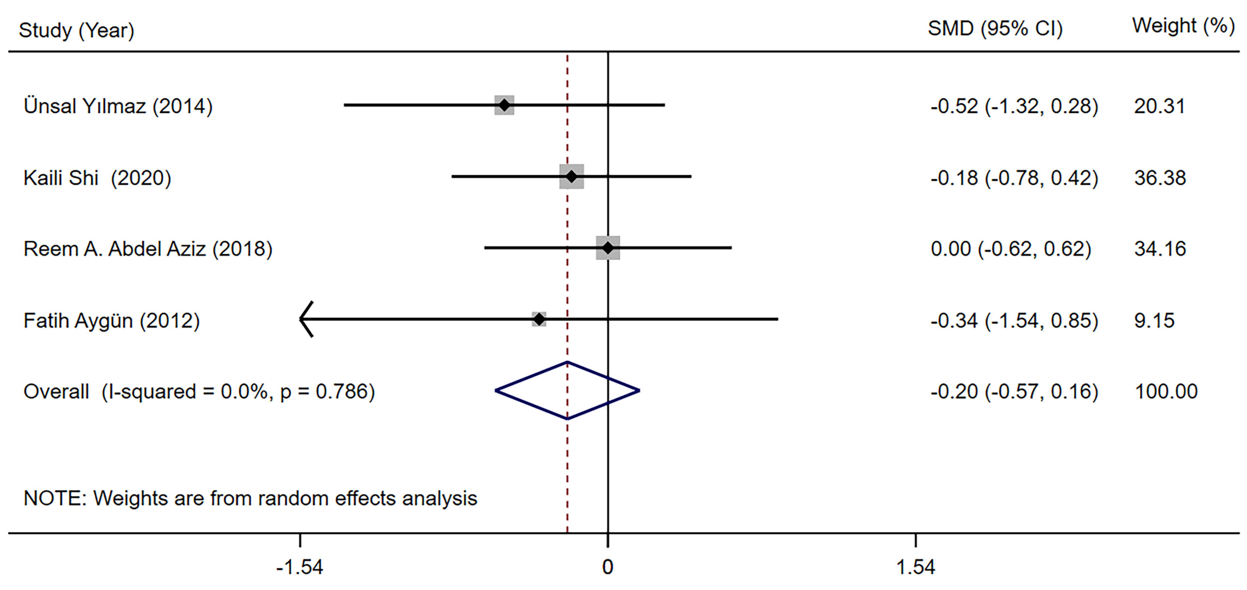

Supplementary Appendix S2. A forest plot of the effect on thyroid stimulating hormone (TSH) levels of levetiracetam (LEV) vs. oxcarbazepine (OXC) before monotherapy in children with epilepsy.

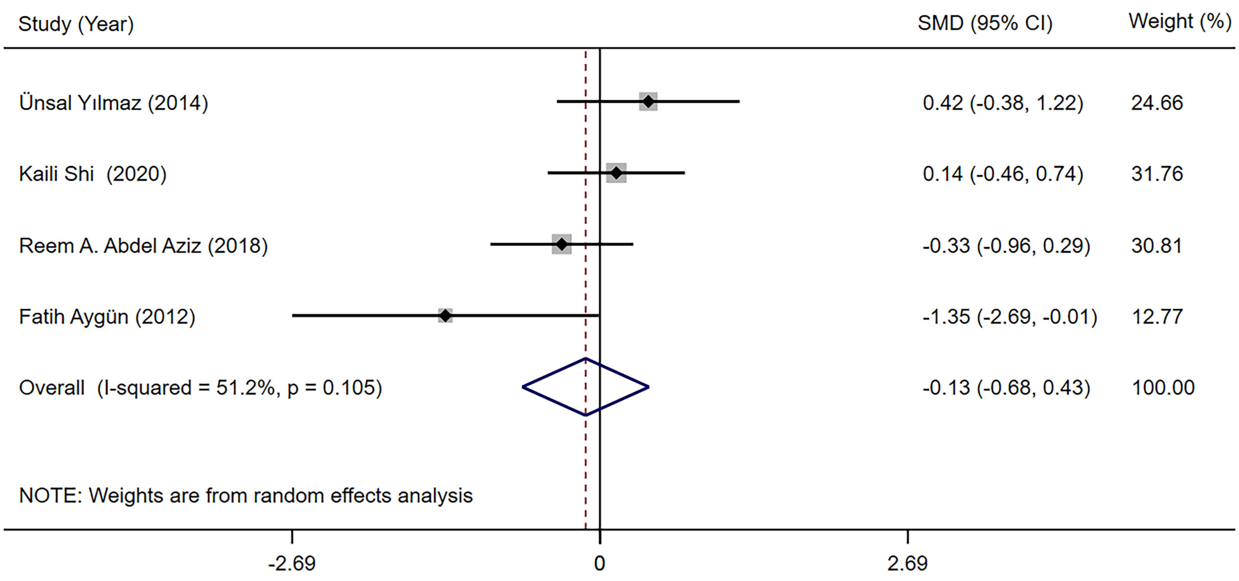

Supplementary Appendix S3. A forest plot of the effect on free thyroxine (fT4) levels of levetiracetam (LEV) vs. oxcarbazepine (OXC) before monotherapy in children with epilepsy.
